# Potential of Icariin Metabolites from *Epimedium koreanum* Nakai as Antidiabetic Therapeutic Agents

**DOI:** 10.3390/molecules22060986

**Published:** 2017-06-13

**Authors:** Da Hye Kim, Hyun Ah Jung, Hee Sook Sohn, Jin Woong Kim, Jae Sue Choi

**Affiliations:** 1 Department of Food and Life Science, Pukyong National University, Busan 48513, Korea; md2253@naver.com; 2 Department of Food Science and Human Nutrition, Chonbuk National University, Jeonju 54896, Korea; hssohn@jbnu.ac.kr; 3 College of Pharmacy, Seoul National University, Seoul 08826, Korea; jwkim@snu.ac.kr

**Keywords:** *Epimedium koreanum* Nakai, icariin metabolite, PTP1B, α-glucosidase, molecular docking simulation

## Abstract

The therapeutic properties of *Epimedium koreanum* are presumed to be due to the flavonoid component icariin, which has been reported to have broad pharmacological potential and has demonstrated anti-diabetic, anti-Alzheimer’s disease, anti-tumor, and hepatoprotective activities. Considering these therapeutic properties of icariin, its deglycosylated icaritin and glycosylated flavonoids (icaeriside II, epimedin A, epimedin B, and epimedin C) were evaluated for their ability to inhibit protein tyrosine phosphatase 1B (PTP1B) and α-glucosidase. The results show that icaritin and icariside II exhibit potent inhibitory activities, with 50% inhibition concentration (IC_50_) values of 11.59 ± 1.39 μM and 9.94 ± 0.15 μM against PTP1B and 74.42 ± 0.01 and 106.59 ± 0.44 μM against α-glucosidase, respectively. With the exceptions of icaritin and icariside II, glycosylated flavonoids did not exhibit any inhibitory effects in the two assays. Enzyme kinetics analyses revealed that icaritin and icariside II demonstrated noncompetitive-type inhibition against PTP1B, with inhibition constant (*K*_i_) values of 11.41 and 11.66 μM, respectively. Moreover, molecular docking analysis confirmed that icaritin and icariside II both occupy the same site as allosteric ligand. Thus, the molecular docking simulation results were in close agreement with the experimental data with respect to inhibition activity. In conclusion, deglycosylated metabolites of icariin from *E. koreanum* might offer therapeutic potential for the treatment of type 2 diabetes mellitus.

## 1. Introduction

Diabetes mellitus (DM) is considered to be one of the foremost challenges of the 21st century. This disease is characterized by a group of metabolic disorders of heterogeneous etiology and is augmented by hyperglycemia resulting from defects in insulin action and secretion. Chronic hyperglycemia in DM is involved in long-term damage, dysfunction, and failure of various organs, especially the eyes, kidneys, nerves, heart, and blood vessels. Diabetes mellitus is a result of insulin deficiency, which is often combined with insulin resistance [1]. DM is prevalent throughout the world, and its incidence is rapidly increasing in most countries [2]. Insulin resistance, a hallmark of type 2 DM (T2DM), results from disequilibrium in auto-phosphorylation of the insulin receptor (IR) and tyrosine kinase activity. Protein tyrosine phosphatase 1B (PTP1B) interacts with the activated IR and with insulin receptor substrate (IRS) proteins, after which it dephosphorylates tyrosine residues on the IR and IRS proteins [3]. PTP1B has been suggested to be a negative regulator of insulin metabolism in vitro. Extensive studies involving genetics and molecular approaches have been conducted and have demonstrated that overexpression of PTP1B results in insulin resistance [4,5]. Therefore, inhibitors of PTP1B are predicted to be useful for the prevention and treatment of T2DM and might also be promising insulin-sensitive drug targets. Another therapeutic approach for decreasing postprandial hyperglycemia is to prevent absorption of carbohydrates after food uptake. Polysaccharide complexes are hydrolyzed to dextrin or oligosaccharides by amylase and are further hydrolyzed to glucose by α-glucosidase. Next, they are absorbed into the intestinal epithelium and enter the blood circulation [6,7]. α-Glucosidase belongs to a group of enzymes consisting of exo-type α-glucosidic *O*-linkage-hydrolases that release D-glucose from the non-reducing substrate end [8]. α-Glucosidase inhibitors such as acarbose, miglitol, and voglibose are generally used to treat patients with diabetes. These inhibitors delay the absorption of carbohydrates from the small intestine. Thus, α-glucosidase inhibitors lower postprandial blood glucose and insulin levels [9]. However, these inhibitors are associated with undesirable side effects, including liver toxicity, abdominal cramping, flatulence, and diarrhea [10]. Since natural α-glucosidase inhibitors are not associated with any adverse effects, they have generated much interest for the treatment of DM. Therefore, pharmacological interventions aimed at preventive and therapeutic inhibition of α-glucosidase are a potential strategy for treating diabetes.

Epimedii Herba (EH), also referred to as “Yin Yang Huo” or Horny Goat Weed, has been used in traditional Chinese medicine (TCM) to treat impotence, female infertility, dysuria, rheumatic arthritis, geriatric depression, and angina pectoris, among other conditions [11,12]. For centuries, it has been known that sheep and goats who consume *Epimedium* species exhibit higher frequencies of coitus. *Epimedium* plants are also referred to as “nine leaves on three stems” (San Zhi Jiu Ye Cao) in China, due to the special characteristics of the aerial parts of *Epimedium* species. Specifically, every *Epimedium* plant has only three stems and only three leaves on each stem [13]. The aerial parts of *E. koreanum* Nakai are widely used in traditional Chinese and Korean herbal medicine [14]. A wide range of pharmacological and biological activities of *E. koreanum* Nakai has been reported. For instance, *E. koreanum* extract has been reported to have anti-diabetic activity [15], to regulate sexual behavior [16,17], and to have antiviral activity [18]. In addition, *E. koreanum* contains a number of constituents including icariin, icariside II, icaritin, epimedoside A–E, epimedin A–C, ikarisoside A–E, syringaresinol, and icariresinol, all of which are flavonoid glycosides or lignans [19,20,21]. Moreover, icariin, the major component of *E. koreanum*, has been reported to have anti-hepatotoxic [22], anti-tumor, and anti-inflammatory activities [23]. Other studies have reported that icariin has bioactivities such as increasing vasodilation [24], improving dysfunction in spinal cord injury [25,26], ameliorating diabetic retinopathy [27], and neuroprotective effects [28]. Moreover, icariside II has potent anti-tumor activity [29], anti-hepatotoxic activity [30], and attenuates streptozotocin-induced cognitive deficits [31]. Icaritin was also reported to have anti-cancer activity [32,33] and to exert neuroprotective effects [34]. Furthermore, Zhang et al. [35] reported that epimediphine has anti-AChE inhibitory activity. Despite the wide pharmacological potential of *E. koreanum*, no systematic investigation has yet examined the possibility of developing personalized anti-DM drug candidates from *E. koreanum*. This study proposes a prospective strategy for the identification of promising anti-DM compounds from *E. koreanum* by using enzyme kinetics and molecular docking approaches.

## 2. Results

### 2.1. Abilities of the MeOH Extract of the Aerial Parts of E. koreanum and Its Solvent-Soluble Fractions to Inhibit PTP1B and α-Glucosidase 

**Figure 1:**
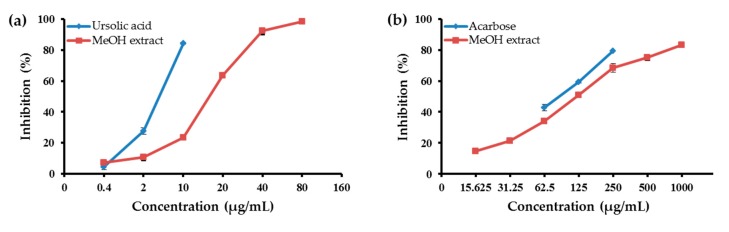
(**a**) Protein tyrosine phosphatase1B (PTP1B); and (**b**) α-glucosidase inhibitory activities of the MeOH extract from aerial parts of *E. koreanum*. Error bar indicates standard error of the mean (SEM).

To determine the anti-diabetic potential of the MeOH extract of the aerial parts of E. koreanum and its soluble fractions, their abilities to inhibit PTP1B and α-glucosidase were evaluated. As shown in Table 1, the CH_2_Cl_2_ fraction strongly inhibited PTP1B at the tested concentrations, with an 50% inhibition concentration (IC_50_) value of 3.51 ± 0.05 μg/mL. For comparison, the positive control ursolic acid had an IC_50_ value of 5.54 ± 0.13 μg/mL. The MeOH extract of the aerial parts of E. koreanum significantly inhibited PTP1B activity in a dose-dependent manner, with an IC_50_ value of 18.83 ± 0.14 μg/mL (Figure 1). Moreover, the EtOAc, n-BuOH, and H_2_O fractions moderately inhibited PTP1B activity, with IC_50_ values of 27.09 ± 0.30, 14.44 ± 0.68, and 38.36 ± 1.29 μg/mL, respectively. The abilities of the MeOH extract and its fractions to inhibit α-glucosidase are shown in Table 1. Among the four solvent-soluble fractions, the CH_2_Cl_2_ and EtOAc fractions were the most potent inhibitors of α-glucosidase, with IC_50_ values of 47.69 ± 0.32 and 75.87 ± 0.38 μg/mL, respectively. For comparison, the positive control acarbose had an IC_50_ value of 87.83 ± 1.08 μg/mL. In contrast, the MeOH extract and two fractions thereof (*n*-BuOH and H_2_O) moderately inhibited α-glucosidase activity, with IC_50_ values of 120.75 ± 3.06, 138.90 ± 0.02, and 310.01 ± 1.29 μg/mL, respectively.

### 2.2. Abilities of the Individual Compounds to Inhibit PTP1B and α-Glucosidase Activity

**Figure 2:**
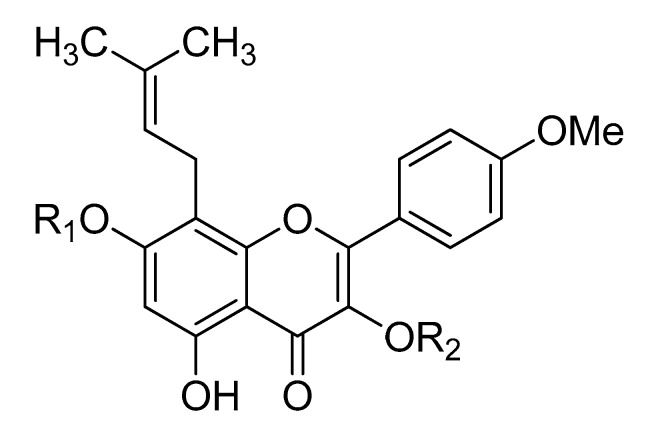
Chemical structures of the compounds.

To identify the active compounds of *E. koreanum* responsible for its potent anti-diabetic activity, the abilities of the compounds (Figure 2) to inhibit PTP1B and α-glucosidase activity were evaluated. As shown in Table 2, icaritin and icariside II were the most potent inhibitors, with IC_50_ values of 11.59 ± 1.39 and 9.94 ± 0.15 μM, respectively. For comparison, ursolic acid had an IC_50_ of 8.24 ± 0.03 μM. However, none of the other compounds inhibited PTP1B at any of the tested concentrations. Among the tested compounds, icaritin and icariside II were the strongest inhibitors of α-glucosidase, with IC_50_ values of 74.42 ± 0.01 and 106.59 ± 0.44 μM, respectively. For comparison, acarbose had an IC_50_ value of 101.16 ± 3.69 μM. In contrast, epimedin A, epimedin B, and epimedin C only moderately inhibited α-glucosidase activity, with IC_50_ values of 444.09 ± 3.80, 451.78 ± 5.36 and 403.87 ± 1.53 μM, respectively. However, icariin did not inhibit α-glucosidase activity at any of the tested concentrations.

### 2.3. Enzyme Kinetics Analysis of PTP1B Inhibition by Selected Compounds

**Figure 3:**
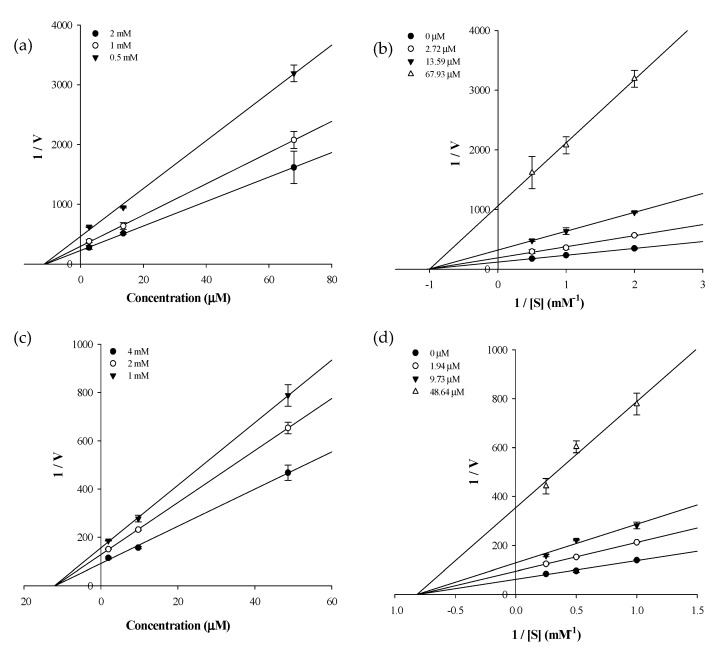
Dixon and Lineweaver–Burk plots of the inhibition of PTP1B by icaritin and icariside II. The results showed the effects of the presence of different concentrations of the substrate for: (**a**) icaritin; and (**c**) icariside II; and the effect of the presence of different concentration of: (**b**) icaritin; and (**d**) icariside II.

Since the PTP1B activity assay revealed that two of the tested compounds (icaritin and icariside II) were active, these compounds were further analyzed to establish the compound-substrate, *p*-nitrophenyl phosphate (*p*NPP) relationships for PTP1B. The inhibition type and inhibition constant (*K*_i_) of the two compounds were determined using Lineweaver–Burk and Dixon plots. Each line of the inhibitors penetrate the same point at the x-intercept, indicating noncompetitive-type inhibition, and the *x*-axis represent-K*_i_*when 1/V = 0. Therefore, icaritin and icariside II are noncompetitive-type inhibitors of PTP1B (Figure 3). The Dixon plot is an established method for determining the enzyme inhibition type and the *K*_i_ value for an enzyme-inhibitor complex, where the value of the *x*-axis indicates *K*_i_. As shown in Table 2, the *K*_i_ values of icaritin and icariside II were 11.41 and 11.66 μM, respectively.

### 2.4. Molecular Docking Simulation of PTP1B Inhibition

**Figure 4:**
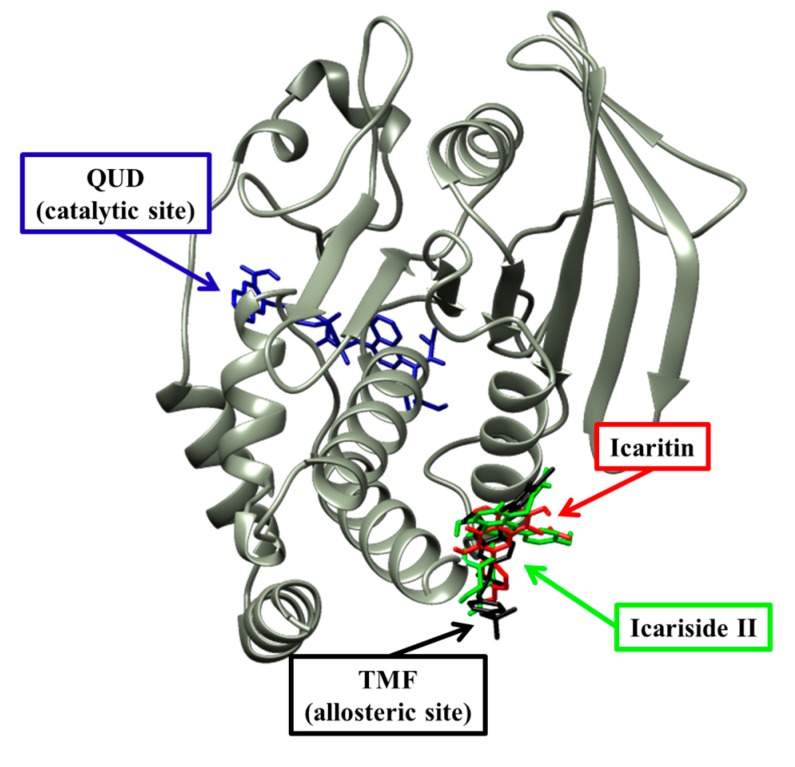
Molecular docking of PTP1B inhibition by compounds (compound **23**, compound **2**, icaritin and icariside II).

**Figure 5:**
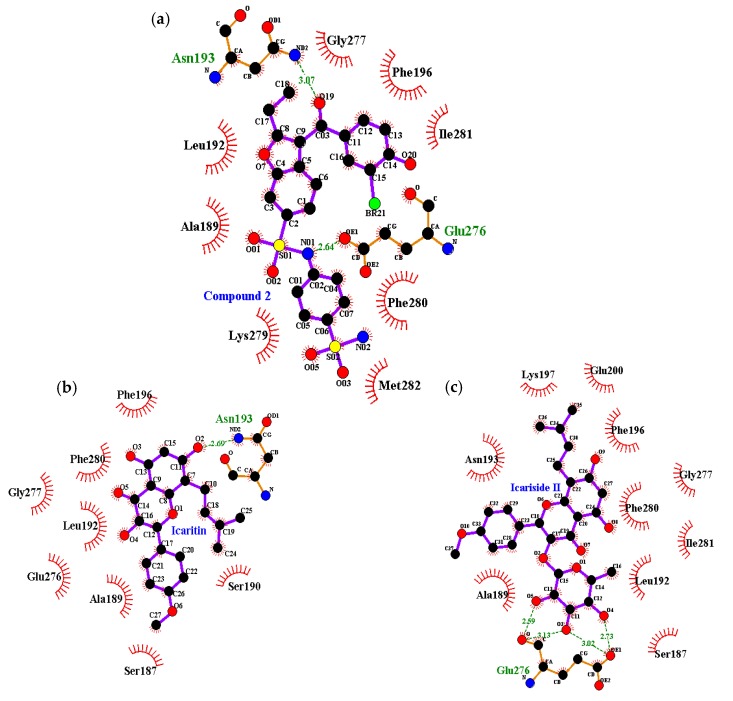
Molecular docking models for PTP1B inhibition of: (**a**) compound **2**; (**b**) icaritin; and (**c**) icariside II.

Next, a molecular docking simulation study was performed to validate the mechanism of interaction between the two compounds and the active site of PTP1B. Specifically, 3D docking of the active compounds (icaritin and icariside II) was simulated. For this simulation, 3-({5-[(*N*-acetyl-3-{4-[(carboxycarbonyl)(2-carboxyphenyl)amino]-1-naphthyl}-l-alanyl)amino]pentyl}oxy)-2-naphthoic acid (positive ligand of the PTP1B catalytic model, compound **23**) and 3-(3,5-dibromo-4-hydroxy-benzoyl)-2-ethyl-benzofuran-6-sulfonic acid (4-sulfamoyl-phenyl)-amide (positive ligand of the allosteric model, compound **2**) were considered as the standard ligands to which the orientation of the tested compounds were compared. Docking analyses were conducted to determine the extent of agreement of the structure-activity compound relationships with the obtained experimental data, as well as to evaluate binding site-directed inhibition of PTP1B. The AutoDock 4.2 program [36] uses a semi-empirical free energy force field to predict the protein–ligand binding complexes of a known structure and the binding energies for the bound state. The results of the docking simulations of the four compounds are shown in Table 3 and Figure 4. The PTP1B-icaritin complex had a binding energy of −6.24 kcal/mol and one hydrogen bond interaction with residue Asn193. The nitrogen group of Asn193 was involved in a strong hydrogen bond with the hydroxyl group of icaritin; this bond had a distance of 2.69 Å. Hydrophobic interactions were also predicted between Ser187, Ala189, Ser190, Leu192, Phe196, Glu276, Gly277, and Phe280 (Figure 5). These hydrophobic interactions are important for strengthening the protein–ligand interaction and positioning icaritin in the active pocket to inhibit PTP1B activity. The binding energy of the PTP1B-icariside II complex was −8.77 kcal/mol, and four hydrogen bonds interacted with Glu276. The oxygen groups of the Glu276 residue were involved in the hydrogen bond interaction with the hydroxyl groups of icariside II; the bond distances ranged from 2.59 to 3.13 Å. In addition, hydrophobic interactions were observed between Ser187, Ala189, Leu192, Asn193, Phe196, Lly197, Glu200, Gly277, Phe280, and Ile281 (Figure 5c). Therefore, hydrogen bonds and hydrophobic interactions with PTP1B might be associated with substrate hydrolysis. The molecular docking simulation results corresponded well with the experimental data with respect to the abilities of the different compounds to inhibit PTP1B.

## 3. Discussion

DM is a major community-level concern. Epidemiological studies have revealed that the prevalence of DM continues to increase, having almost attained epidemic proportions worldwide. As of 2015, more than 415 million adults had DM. This number is expected to grow to 642 million by 2040 [37]. Over the past decade, the growing epidemic of DM and its associated complications have presented both challenges and opportunities. Significant therapeutic developments have been made, and many novel chemical compounds have been tested for their ability to treat diabetes; however, the long-term safety and efficacy of these chemicals are not yet established. Thus, finding new natural drug candidates for DM, with no or reduced side effects, is an important goal for improving treatment and prevention of diabetes. PTP1B is a negative regulator of the insulin metabolic pathway, and α-glucosidase promotes the absorption of carbohydrates into blood vessels by hydrolyzing oligosaccharides to glucose. Despite the identification of many potent compounds [38,39,40,41], it is still essential to search for new inhibitors with better safety and high efficacy. *E. koreanum* is used in TCM to treat impotence, female infertility, dysuria, rheumatic arthritis, geriatric depression, and angina pectoris [11,12]. Despite the broad pharmacological effects of *E. koreanum*, no systematic investigation prior to the present study investigated the possibility of identifying anti-DM drug candidates from *E. koreanum*.

As summarized in Table 1, the anti-DM activity of the MeOH extract was examined via in vitro PTP1B and α-glucosidase activity assays. Similarly, the effects of the CH_2_Cl_2_, EtOAc, *n*-BuOH, and H_2_O fractions were assessed in the same assays. The results showed that the MeOH extract of the aerial parts of *E. koreanum* inhibited both PTP1B and α-glucosidase in a dose-dependent manner (Figure 1). Of the four solvent-soluble fractions, the CH_2_Cl_2_ fraction showed the most potent inhibition of PTP1B and α-glucosidase. In addition, icaritin and icariside II showed inhibitory potential, with IC_50_ values of 11.59 ± 1.39 and 9.94 ± 0.15 μM against PTP1B and 74.42 ± 0.01 and 106.59 ± 0.44 μM against α-glucosidase, respectively. In conclusion, icaritin and icariside II, two deglycosylated metabolites of icariin, could potentially play therapeutically important roles as PTP1B and α-glucosidase inhibitors. In previous studies, *E. koreanum* extract was pharmacologically and biologically evaluated. These studies revealed that the extract exhibits osteoblastic proliferation stimulating activity [42], affects kidney-yang deficiency syndrome [43], and possesses estrogenic activity [44]. In addition, Oh et al. [15] reported that EH extract has significant anti-diabetic activity in rats with streptozotocin-induced diabetes. Furthermore, epimedin A–C did not inhibit α-glucosidase, similar to the findings of a previous study [45]. Moreover, icaritin and icariside II from *E. koreanum* also showed potent anti-diabetic activities. This study is the first to report the potencies of these compounds.

To establish the modes of inhibition of the two compounds with high inhibitory activity (icaritin and icariside II), enzyme kinetics and molecular docking studies were performed. The Lineweaver–Burk and Dixon plots revealed that both of the active compounds were non-competitive inhibitors of PTP1B because they decreased the *V*_max_ values without changing the *K*_m_ values. To predict binding sites and affinities, molecular docking simulations were performed. The best docked conformation was selected based on binding affinity, hydrogen bonds, and hydrophobic interactions. Amino acid residues of enzymes interact with compounds via H-bonds and hydrophobic interactions [46]. Computational docking simulations are one method for comprehensively understanding the mechanism behind active site binding interactions [47]. To avoid potential bottlenecks in the development of PTP1B inhibitors, much attention has focused on allosteric PTP1B inhibitors because allosteric inhibitors have higher specificity, fewer side effects, and lower toxicity [48,49]. The allosteric site is located on the C-terminal domain of PTP1B and is bordered by helices α3, α6, and α7, which constitute a hydrophobic pocket for allosteric inhibitors [50]. The WPD loop, which contributes to the recognition of acids/bases via Asp181, is the primary determinant for the binding of catalytic substrates in PTP1B. Moreover, allosteric inhibition of PTP1B was shown to be associated with closure of the catalytic WPD loop [51,52]. In the presence of allosteric inhibitors, the WPD loop adopts a different conformation compared to that in the Apo form of PTP1B [50]. Compound **2**, which is a positive inhibitor of the allosteric PTP1B model, forms two hydrogen bonds with allosteric sites of PTP1B (e.g., Asp193 and Glu276) (Table 3 and Figure 5a). The hydroxyl group of icaritin interacted with PTP1B, forming a single hydrogen bond with Asn193 with a binding energy of −6.24 kcal/mol (Figure 5b). Icariside II showed a lower binding score to PTP1B than icaritin, indicating high affinity. The Glu276 residue interacted with the hydroxyl groups of the α-l-rhamnose moiety of icariside II by forming four hydrogen bonds (Figure 5c). These hydrophobic interactions contributed to the higher binding affinity between PTP1B and the compounds. The molecular docking simulation results agreed well with the in vitro PTP1B activity assay results, where icariside II and icaritin were potent PTP1B inhibitors (Table 2).

Recently, Wu et al. [53] demonstrated that icariin is hydrolyzed to icariside II and icaritin by human intestinal microflora. Other studies have reported that icariin ameliorates streptozotocin-induced diabetic retinopathy [27], reduces mitochondrial oxidative stress injury in the hearts of diabetic rats [54], and protects mice from cisplatin-induced acute renal injury [55]. Furthermore, icariside II has been shown to ameliorate diabetic nephropathy in rats with streptozotocin-induced diabetes [56], and icaritin has been reported to have anti-inflammatory and anti-oxidative effects in rats under stress [57]. The findings of the present study indicate that the in vivo effects of icariin can be attributed to the activity of two of its metabolites, icariside II and icaritin.

## 4. Materials and Methods

### 4.1. Plant Materials

The aerial parts of *Epimedium koreanum* were collected from the Medicinal Herb Garden, Seoul, Korea, in November 2008 and confirmed by Professor Jinwoong Kim at the College of Pharmacy, Seoul National University. A voucher specimen (no. 20081105) was deposited in the authorized laboratory.

### 4.2. General Experimental Procedures

^1^H-NMR and ^13^C-NMR spectra were measured using a JEOL JNM ECP-400 spectrometer (JEOL, Tokyo, Japan) at 400 and 100 MHz, respectively. Samples were dissolved in dimethyl sulfoxide (DMSO-*d*_6_) or methyl alcohol (CD_3_OD). Bioassays, including PTP1B and α-glucosidase activity assays, were conducted using a microplate spectrophotometer (Molecular Devices, VERSA max, CA, USA).

### 4.3. Chemicals and Reagents

*p*NPP, ethylenediamine tetra acetic acid (EDTA), yeast α-glucosidase, *p*-nitrophenyl α-d-glucopyranoside (*p*NPG) and acarbose were purchased from Sigma-Aldrich Co. (St. Louis, MO, USA). PTP1B (human recombinant) was purchased from Biomol International LP (Plymouth Meeting, PA, USA), and dithiothreitol (DTT) was purchased from Bio-Rad Laboratories (Hercules, CA, USA).

### 4.4. Extraction and Fractionation

The dried aerial parts of *E. koreanum* (2.4 kg) were extracted with MeOH (5 L × 3 times) for 3 h under reflux. After filtration, the entire filtrate was concentrated to dryness in vacuo at 40 °C, yielding the MeOH extract (655.2 g).

### 4.5. Isolation of Prenylated Flavonoids from E. koreanum

Repeated chromatography of the CH_2_Cl_2_ fraction over a Si gel column with a CH_2_Cl_2_-MeOH gradient followed by a Sephadex LH-20 column was used to isolate icaritin (37 mg) [58]. Further chromatography of the *n*-BuOH fraction over a silica gel column with CH_2_Cl_2_–MeOH–H_2_O (10:1:0.1) yielded 10 subfractions (EKB1-10). EKB2 (210 mg) was chromatographed on Si and RP-18 gel columns using EtOAc–MeOH–H_2_O (200:2:1) and a 60% MeOH solvent system to obtain icariside II (27 mg) [59]. EKB4 (3.1143 g) was recrystallized and further purified with a CH_2_Cl_2_–MeOH–H_2_O (7:1:0.1) solvent system to afford icariin (795 mg) [60]. EKB7 (929.6 mg) was chromatographed on a RP-18 gel column and eluted with a 50% MeOH solvent system to yield 7 fractions (EKB71-77). EKB75 was further subjected to Si column chromatography with EtOAc–MeOH–H_2_O (21:3:2) to yield epimedin B (6 mg) and epimedin A (107.6 mg) [61]. EKB76 was chromatographed on a Si column with an EtOAc–MeOH–H_2_O (21:3:3) solvent system to give epimedin C (47.7 mg) [61]. The chemical structures of the isolated compounds (Figure 2) were characterized on the basis of ^1^H- and ^13^C-NMR spectral analysis, and comparisons with published spectral data. The purity of all of the isolated compounds was over 98%. 

### 4.6. PTP1B Activity Assay

The abilities of the aerial parts of *E. koreanum* and their constituents to inhibit human recombinant PTP1B activity were evaluated using *p*NPP as a substrate [62]. Briefly, 40 µL of recombinant PTP1B enzyme [0.5 units diluted with a PTP1B reaction buffer containing 50 mM citrate (pH 6.0), 0.1 M NaCl,1 mM EDTA, and 1 mM DTT] was added to each well of a 96-well plate (final volume 100 µL), with or without a test sample. Then, 50 μL of 2 mM *p*NPP dissolved in PTP1B reaction buffer was added. After incubation at 37 °C for 20 min in the dark, the reaction was terminated by addition of 10 M NaOH. The amount of *p*-nitrophenylate produced by enzymatic dephosphorylation of *p*NPP was determined by measuring the absorbance at 405 nm using a microplate spectrophotometer (Molecular Devices). Non-enzymatic dephosphorylation of *p*NPP was corrected by measuring the increase in absorbance at 405 nm obtained in the absence of PTP1B. Ursolic acid was used as a positive control. The percent inhibition was calculated as {(A_C_ − A_S_)/A_C_} × 100, where A_C_ is the absorbance of the control, and A_S_ is the absorbance of the sample. The IC_50_ is expressed as the mean ± SEM of triplicate experiments.

### 4.7. α-Glucosidase Activity Assay

α-Glucosidase enzymatic activity was assayed spectrophotometrically as described previously [63]. Briefly, 60 μL of reaction mixture containing 20 μL of 100 mM phosphate buffer (pH 6.8), 20 μL of 2.5 mM *p*NPG, and 20 μL of sample (final concentration ranging from 20 to 300 μM, dissolved in 10% DMSO) was added to each well. After incubation at 37 °C for 5 min in the dark, 20 μL of yeast α-glucosidase (0.2 U/mL) in 10 mM phosphate buffer (pH 6.8) was added. The plate was then incubated at 37 °C for 15 min in the dark, after which the reaction was stopped by the addition of 80 μL of 0.2 M sodium carbonate solution. The absorbance was immediately recorded at 405 nm using a microplate spectrophotometer (Molecular Devices). The control consisted of the same reaction mixture but with the sample solution replaced by an equivalent volume of phosphate buffer. Acarbose dissolved in 10% DMSO was used as a positive control. The inhibition percentage (%) was obtained using the same equation as in the PTP1B enzymatic assay.

### 4.8. Enzyme Kinetics Analysis of PTP1B

In order to determine the inhibition mechanism, two complementary kinetics methods were used: Lineweaver–Burk and Dixon plots [64,65,66]. The level of enzymatic inhibition at various concentrations of test sample was evaluated by monitoring the effect of different substrate concentrations in Dixon plots (single reciprocal plots). Dixon plots for PTP1B inhibition were obtained in the presence of different concentrations of *p*NPP. Using Lineweaver–Burk plots (double reciprocal plots), the inhibition modes of the compounds were determined at various concentrations of *p*NPP with different concentrations of compounds. The enzymatic procedures were the same as for the PTP1B assay methods. The types of inhibition were investigated by interpreting the Dixon plots, in which the value of the *x*-axis indicates *K*_i_.

### 4.9. Molecular Docking Simulation of PTP1B Inhibition

The X-ray crystallographic structure of PTP1B complexed with its selective inhibitor 3-(3,5-dibromo-4-hydroxy-benzoyl)-2-ethyl-benzofuran-6-sulfonic acid (4-sulfamoyl-phenyl)-amide (compound **2**) (PDB ID: 1T49) and the 3D structure of its catalytic inhibitor 3-({5-[(*N*-acetyl-3-{4-[(carboxycarbonyl)(2-carboxyphenyl)amino]-1-naphthyl}-l-alanyl)amino]pentyl}oxy)-2-naphthoic acid (compound **23**) were obtained from the RCSB Protein Data Bank [67,68] website. The resolution of the crystal structure was 1.9 Å [69], and the PubChem Compound (NCBI) CID was 447410. The bound PTP1B inhibitor and water molecules were removed from the structure for the docking simulation using Accelrys Discovery Studio 4.1 software (Accelrys, Inc. San Diego, CA, USA). The 3D structures of icaritin and icariside II were obtained from PubChem Compound (NCBI), corresponding to compound CIDs of 5318980 and 44587252, respectively, and protonated (pH 7.0) using the MarvinSketch program (ChemAxon, Budapest, Hungary). Automated docking simulation was performed using AutoDock Tool (ADT) to assess the appropriate binding orientations and conformations of the PTP1B with the different compounds. A Lamarkian genetic algorithm method implemented in AutoDock 4.2 was employed. For docking calculations, Gasteiger charges were added by default, the rotatable bonds were set by the ADTs, and all torsions were allowed to rotate. Grid maps were generated by the Autogrid program; the grid box size was 80 × 80 × 80 and had a default spacing of 0.375 Å. The X, Y, and Z centers were 51, 19.034, and 14.048, respectively. The docking protocol for rigid and flexible ligand docking consisted of 10 independent genetic algorithms, whereas ADT default settings were used for the other parameters. Binding PTP1B residues and their corresponding binding affinity scores were regarded as the best molecular interactions. The results were analyzed using UCSF Chimera [70], while the hydrogen bonds and van der Waals interaction residues were visualized by Ligplot 1.4.5.

### 4.10. Statistics

All results are expressed as the mean ± SEM of triplicate samples. Statistical significance was analyzed using one-way ANOVA and Student’s *t*-test (Systat Inc., Evanston, IL, USA).

## 5. Conclusions

The results of the current study indicate that the aerial parts of *E. koreanum* and their constituents possess anti-DM properties. Comparative evaluations of these activities of *E. koreanum* might be useful in the development of therapeutic agents for treating patients with DM. Among the tested compounds, icaritin and icariside II (two deglycosylated metabolites of icariin) significantly inhibited PTP1B. Furthermore, enzyme kinetics analysis and molecular docking simulations supported these results. Thus, approaches combining computational work with experimental validation are useful for developing new natural drug candidates against diabetes. These active candidates have significant potential as drug candidates for treating DM. Further investigations of the bioactivity of these natural products are needed to determine the precise therapeutic and/or preventive potential of *E. koreanum* and its constituents in vivo.

## Figures and Tables

**Table:** 

Icaritin	R_1_ = H	R_2_ = H
Icariside II	R_1_ = H	R_2_ = rha
Icariin	R_1_ = glc	R_2_ = rha
Epimedin A	R_1_ = glc	R_2_ = rha-glc
Epimedin B	R_1_ = glc	R_2_ = rha-xyl
Epimedin C	R_1_ = glc	R_2_ = rha-rha

**Table 1:** Abilities of the MeOH extract of *Epimedium koreanum* and its fractions to inhibit protein tyrosine phosphatase1B (PTP1B) and α-glucosidase.

Sample	Yield (g)	IC_50_ (μg/mL) ^a^	
		PTP1B	α-Glucosidase
MeOH extract	665.2	18.83 ± 0.14	120.75 ± 3.06
CH_2_Cl_2_ fraction	56.94	3.51 ± 0.05	47.69 ± 0.32
EtOAc fraction	78.20	27.09 ± 0.30	75.87 ± 0.38
*n*-BuOH fraction	237.12	14.44 ± 0.68	138.90 ± 0.02
H_2_O fraction	268.06	38.36 ± 1.29	310.01 ± 1.29
Ursolic acid ^b^	-	5.54 ± 0.13	
Acarbose ^b^	-		87.83 ± 1.08

^a^ The 50% inhibitory concentration (IC_50_) values (μg/mL) were calculated from a log dose inhibition curve and are expressed as mean ± standard error of the mean (SEM) of triplicate experiments. ^b^ Used as positive control.

**Table 2:** Abilities of *Epimedium koreanum-*derived compounds to inhibit protein PTP1B and α-glucosidase.

Compound	PTP1B			α-Glucosidase
	IC_50_ (μM) ^a^	*K*_i_ (μM) ^b^	Inhibition Type ^c^	IC_50_ (μM) ^a^
Icaritin	11.59 ± 1.39	11.41	Non-competitive	74.42 ± 0.01
Icariside II	9.94 ± 0.15	11.66	Non-competitive	106.59 ± 0.44
Icariin	>300	-	-	>300
Epimedin A	>300	-	-	>300
Epimedin B	>300	-	-	>300
Epimedin C	>300	-	-	>300
Ursolic acid ^d^	8.24 ± 0.30			
Acarbose ^d^				101.16 ± 3.69

^a^ The IC_50_ values (μM) were calculated from a log dose inhibition curve and are expressed as mean ± SEM of triplicate experiments. ^b^ Inhibition constant (*K*_i_) value is determined by the Dixon plot. ^c^ Determined by the Lineweaver–Burk plot. ^d^ Used as positive control.

**Table 3:** Binding site residues and docking scores of compounds with PTP1B as determined by AutoDock 4.2.

Compound	Binding Energy ^a^ (kcal/mol)	No. of H-Bonds	H-Bond Interacting Residues ^b^	Van der Waals Bond Interacting Residues ^c^
Compound **23** ^d^ (catalytic inhibitor)	−11.23	11	Tyr46, Asp48, Arg24, Ser216, Ala217, Arg221, Arg254, Gln262	Ser28, Val49, Lly116, Lly120, Cys215, Ile219, Gly220, Met258, Gly259
Compound **2** ^d^ (allosteric inhibitor)	−10.98	2	Asn193, Glu276	Phe196, Gly277, Phe280, Ile281, Met282, Lys279, Ala189, Leu192
Icaritin	−6.24	1	Asn193	Ser187, Ala189, Ser190, Leu192, Phe196, Glu276, Gly277, Phe280
Icariside II	−8.77	4	Glu276	Ser187, Ala189, Leu192, Asn193, Phe196, Lys197, Glu200, Gly277, Phe280, Ile281

^a^ Estimated binding free energy of the ligand-receptor complex. ^b,c^ All amino acid residues located 5.0 Å from the original enzyme/compound complex in the AutoDock 4.2 program. ^d^ Compound **23** (3-({5-[(*N*-acetyl-3-{4-[(carboxycarbonyl)(2-carboxyphenyl)amino]-1-naphthyl}-l-alanyl)amino]pentyl}oxy)-2-naphthoic acid) and compound **2** (3-(3,5-dibromo-4-hydroxy-benzoyl)-2-ethyl-benzofuran-6-sulfonic acid (4-sulfamoyl-phenyl)-amide) were used as positive ligands.

## References

[1] The Expert Committee on the Diagnosis and Classification of Diabetes Mellitus (1997). Report of the expert committee on the diagnosis and classification of diabetes mellitus. Diabetes Care.

[2] Parthasarathy R., Ilavarasan R., Karrunakaran C.M. (2009). Antidiabetic activity of Thespesia Populnea bark and leaf extract against streptozotocin induced diabetic rats. Int. J. PharmTech. Res..

[3] Kwon J.H., Chang M.J., Seo H.W., Lee J.H., Min B.S., Na M., Kim J.C., Woo M.H., Choi J.S., Lee H.K. (2008). Triterpenoids and a sterol from the stem-bark of *Styrax japonica* and their protein tyrosine phosphatase 1B inhibitory activities. Phytother. Res..

[4] Elchebly M., Payette P., Michaliszyn E., Cromlish W., Collins S., Loy A.L., Normandin D., Cheng A., Himms-Hagen J., Chan C.C. (1999). Increased insulin sensitivity and obesity resistance in mice lacking the protein tyrosine phosphatase-1B gene. Science.

[5] Liu Z.Q., Liu T., Chen C., Li M.Y., Wang Z.Y., Chen R.S., Wei G.X., Wang X.Y., Luo D.Q. (2015). Fumosorinone, a novel PTP1B inhibitor, activates insulin signaling in insulin-resistance HepG2 cells and shows anti-diabetic effect in diabetic KKAy mice. Toxicol. Appl. Pharmacol..

[6] Shobana S., Sreerama Y.N., Malleshi N.G. (2009). Composition and enzyme inhibitory properties of finger millet (*Eleusine coracana* L.) seed coat phenolics: Mode of inhibition of α-glucosidase and pancreatic amylase. Food Chem..

[7] Choi C.W., Choi Y.H., Cha M.R., Yoo D.S., Kim Y.S., Yon G.H., Hong K.S., Kim Y.H., Ryu S.Y. (2010). Yeast α-glucosidase inhibition by isoflavones from plants of Leguminosae as an in vitro alternative to acarbose. J. Agric. Food Chem..

[8] Chiba S. (1997). Molecular mechanism in α-glucosidase and glucoamylase. Biosci. Biotechnol. Biochem..

[9] Van De Laar F.A., Lucassen P.L., Akkermans R.P., Van De Lisdonk E.H., Rutten G.E., Van Weel C. (2005). α-Glucosidase inhibitors for patients with type 2 diabetes results from a cochrane systematic review and meta-analysis. Diabetes Care.

[10] Etxeberria U., de la Garza A.L., Campión J., Martínez J.A., Milagro F.I. (2012). Antidiabetic effects of natural plant extracts via inhibition of carbohydrate hydrolysis enzymes with emphasis on pancreatic alpha amylase. Expert Opin. Ther. Targets.

[11] Nakashima K., Miyashita H., Yoshimitsu H., Fujiwara Y., Nagai R., Ikeda T. (2016). Two new prenylflavonoids from Epimedii Herba and their inhibitory effects on advanced glycation end-products. J. Nat. Med..

[12] Chen X.J., Tang Z.H., Li X.W., Xie C.X., Lu J.J., Wang Y.T. (2015). Chemical constituents, quality control, and bioactivity of Epimedii Folium (Yinyanghuo). Am. J. Chin. Med..

[13] Wu H., Lien E.J., Lien L.L. (2003). Chemical and pharmacological investigations of Epimedium species: A survey. Prog. Drug Res..

[14] Oh M.H., Houghton P.J., Whang W.K., Cho J.H. (2004). Screening of Korean herbal medicines used to improve cognitive function for anti-cholinesterase activity. Phytomedicine.

[15] Oh T.W., Kang S.Y., Kim K.H., Song M.Y., Park Y.K. (2013). Anti-diabetic effect of medicinal plants used for lower wasting-thirst in streptozotocin-induced diabetic rats. Korea J. Herbol..

[16] Makarova M.N., Pozharitskaya O.N., Shikov A.N., Tesakova S.V., Makarov V.G., Tikhonov V.P. (2007). Effect of lipid-based suspension of *Epimedium koreanum* Nakai extract on sexual behavior in rats. J. Ethnopharmacol..

[17] Kang H.K., Choi Y.H., Kwon H., Lee S.B., Kim D.H., Sung C.K., Park Y.I., Dong M.S. (2012). Estrogenic/antiestrogenic activities of a *Epimedium koreanum* extract and its major components: In vitro and in vivo studies. Food Chem. Toxicol..

[18] Cho W.K., Kim H., Choi Y.J., Yim N.H., Yang H.J., Ma J.Y. (2012). *Epimedium koreanum* Nakai water extract exhibits antiviral activity against porcine epidermic diarrhea virus in vitro and in vivo. Evid. Based Complement. Alternat. Med..

[19] Keum J.H., Han H.Y., Roh H.S., Seok J.H., Lee J.K., Jeong J., Kim J.A., Woo M.H., Choi J.S., Min B.S. (2014). Analysis and stability test of the extracts from Epimedii Herba, Atractylodis Rhizoma Alba and Polygalae Radix for toxicity study. Korea J. Pharmacogn..

[20] Li W.K., Xiao P.G., Pan J.Q. (1998). Complete assignment of ^1^H- and ^13^C-NMR spectra of ikarisoside A and epimedoside C. Magn. Reson. Chem..

[21] Li W.K., Pan J.Q., Lü M.J., Zhang R.Y., Xiao P.G. (1995). A 9,10-dihydrophenanthrene derivate from *Epimedium koreanum*. Phytochemistry.

[22] Lee M.K., Choi Y.J., Sung S.H., Shin D.I., Kim J.W., Kim Y.C. (1995). Antihepatotoxic activity of icariin, a major constituent of *Epimedium koreanum*. Planta Med..

[23] Zhou J., Wu J., Chen X., Fortenbery N., Eksioglu E., Kodumudi K.N., Pk E.B., Dong J., Djeu J.Y., Wei S. (2011). Icariin and its derivative, ICT, exert anti-inflammatory, anti-tumor effects, and modulate myeloid derived suppressive cells (MDSCs) functions. Int. Immunopharmacol..

[24] Xu H.B., Huang Z.Q. (2007). Vasorelaxant effects of icariin on isolated canine coronary artery. J. Cardiovasc. Pharmacol..

[25] Tohda C., Nagata A. (2012). *Epimedium koreanum* extract and its constituent icariin improve motor dysfunction in spinal cord injury. Evid. Based Complement. Alternat. Med..

[26] Zhang L., Shen C., Chu J., Zhang R., Li Y., Li L. (2014). Icariin decreases the expression of APP and BACE-1 and reduces the β-amyloid burden in an APP transgenic mouse model of Alzheimer’s disease. Int. J. Biol. Sci..

[27] Xin H., Zhou F., Liu T., Li G.Y., Liu J., Gao Z.Z., Bai G.Y., Lu H., Xin Z.C. (2012). Icariin ameliorates streptozotocin-induced diabetic retinopathy in vitro and in vivo. Int. J. Mol. Sci..

[28] Chen Y.J., Zheng H.Y., Huang X.X., Han S.X., Zhang D.S., Ni J.Z., He X.Y. (2016). Neuroprotective effects of icariin on brain metabolism, mitochondrial functions, and cognition in triple-transgenic Alzheimer’s disease mice. CNS Neurosci. Ther..

[29] Lin X., Li W.K., Xiao P.G. (1999). Effects of icariside II from *Epimedium koreanum* on tumour cell lines in vitro. Pharm. Pharmacol. Commun..

[30] Cho N.J., Sung S.H., Lee H.S., Jeon M.H., Kim Y.C. (1995). Anti-hepatotoxic activity of icariside II, a constituent of *Epimedium koreanum*. Arch. Pharm. Res..

[31] Yin C., Deng Y., Gao J., Li X., Liu Y., Gong Q. (2016). Icariside II, a novel phosphodiesterase-5 inhibitor, attenuates streptozotocin-induced cognitive deficits in rats. Neuroscience.

[32] Huang X., Zhu D., Lou Y. (2007). A novel anticancer agent, icaritin, induced cell growth inhibition, G 1 arrest and mitochondrial transmembrane potential drop in human prostate carcinoma PC-3 cells. Eur. J. Pharmacol..

[33] Guo Y., Zhang X., Meng J., Wang Z.Y. (2011). An anticancer agent icaritin induces sustained activation of the extracellular signal-regulated kinase (ERK) pathway and inhibits growth of breast cancer cells. Eur. J. Pharmacol..

[34] Wang Z., Zhang X., Wang H., Qi L., Lou Y. (2007). Neuroprotective effects of icaritin against beta amyloid-induced neurotoxicity in primary cultured rat neuronal cells via estrogen-dependent pathway. Neuroscience.

[35] Zhang X., Oh M., Kim S., Kim J., Kim H., Kim S., Houghton P.J., Whang W. (2013). Epimediphine, a novel alkaloid from *Epimedium koreanum* inhibits acetylcholinesterase. Nat. Prod. Res..

[36] Morris G.M., Huey R., Lindstrom W., Sanner M.F., Belew R.K., Goodsell D.S., Olson A.J. (2009). AutoDock 4 and AutoDockTools 4: Automated docking with selective receptor flexibility. J. Comput. Chem..

[37] International Diabetes Federation (IDF) (2015). IDF Diabetes Atlas.

[38] Wang L.J., Jiang B., Wu N., Wang S.Y., Shi D.Y. (2015). Natural and semisynthetic protein tyrosine phosphatase 1B (PTP1B) inhibitors as anti-diabetic agents. RSC Adv..

[39] Bongard R.D., Lepley M., Thakur K., Talipov M.R., Nayak J., Lipinski R.A.J., Bohl C., Sweeney N., Ramchandran R., Rathore R. (2017). Serendipitous discovery of lightinduced (In Situ) formation of an Azo-bridged dimeric sulfonated naphthol as a potent PTP1B inhibitor. BMC Biochem..

[40] Gubiani J.R., Wijeratne E.M., Shi T., Araujo A.R., Arnold A.E., Chapman E., Gunatilaka A.A. (2017). An epigenetic modifier induces production of (10'S)-verruculide B, an inhibitor of protein tyrosine phosphatases by Phoma sp. nov. LG0217, a fungal endophyte of Parkinsonia microphylla. Bioorg. Med. Chem..

[41] Jung H.A., Ali M.Y., Choi J.S. (2016). Promising inhibitory effects of anthraquinones, naphthopyrone, and naphthalene glycosides, from *Cassia obtusifolia* on α-glucosidase and human protein tyrosine phosphatases 1B. Molecules.

[42] Meng F., Xiong Z., Jiang Z., Li F. (2005). Osteoblastic proliferation stimulating activity of *Epimedium koreanum*. Nakai extracts and its flavonol glycosides. Pharm. Biol..

[43] Huang D., Yang J., Lu X., Deng Y., Xiong Z., Li F. (2013). An integrated plasma and urinary metabonomic study using UHPLC–MS: Intervention effects of *Epimedium koreanum* on ‘Kidney-Yang Deficiency syndrome’ rats. J. Pharm. Biomed. Anal..

[44] Islam M.N., Kim U., Kim D.H., Dong M.S., Yoo H.H. (2012). High-performance liquid chromatography-based multivariate analysis to predict the estrogenic activity of an *Epimedium koreanum* extract. Biosci. Biotechnol. Biochem..

[45] Phan M.A.T., Wang J., Tang J., Lee Y.Z., Ng K. (2013). Evaluation of α-glucosidase inhibition potential of some flavonoids from *Epimedium brevicornum*. LWT-Food Sci. Technol..

[46] Kumar K.M., Anbarasu A., Ramaiah S. (2014). Molecular docking and molecular dynamics studies on β-lactamases and penicillin binding proteins. Mol. BioSyst..

[47] Seong S.H., Roy A., Jung H.A., Jung H.J., Choi J.S. (2016). Protein tyrosine phosphatase 1B and α-glucosidase inhibitory activities of *Pueraria lobata* root and its constituents. J. Ethnopharmacol..

[48] Baskaran S.K., Goswami N., Selvaraj S., Muthusamy V.S., Lakshmi B.S. (2012). Molecular dynamics approach to probe the allosteric inhibition of PTP1B by chlorogenic and cichoric acid. J. Chem. Inf. Model..

[49] Huang Z., Mou L., Shen Q., Lu S., Li C., Liu X., Wang G., Li S., Geng L., Liu Y. (2014). ASD v2. 0: Updated content and novel features focusing on allosteric regulation. Nucleic Acids Res..

[50] Li S., Zhang J., Lu S., Huang W., Geng L., Shen Q., Zhang J. (2014). The mechanism of allosteric inhibition of protein tyrosine phosphatase 1B. PLoS ONE.

[51] Lee S., Wang Q. (2007). Recent development of small molecular specific inhibitor of protein tyrosine phosphatase 1B. Med. Res. Rev..

[52] Choi J.S., Ali M.Y., Jung H.A., Oh S.H., Choi R.J., Kim E.J. (2015). Protein tyrosine phosphatase 1B inhibitory activity of alkaloids from Rhizoma Coptidis and their molecular docking studies. J. Ethnopharmacol..

[53] Wu H., Kim M., Han J. (2016). Icariin metabolism by human intestinal microflora. Molecules.

[54] Bao H., Chen L. (2011). Icariin reduces mitochondrial oxidative stress injury in diabetic rat hearts. Zhongguo Zhong Yao Za Zhi.

[55] Ma P., Zhang S., Su X., Qiu G., Wu Z. (2015). Protective effects of icariin on cisplatin-induced acute renal injury in mice. Am. J. Transl. Res..

[56] Tian W., Lei H., Guan R., Xu Y., Li H., Wang L., Yang B., Gao Z., Xin Z. (2015). Icariside II ameliorates diabetic nephropathy in streptozotocin-induced diabetic rats. Drug Des. Devel. Ther..

[57] Zhang W., Xing B., Yang L., Shi J., Zhou X. (2015). Icaritin attenuates myocardial ischemia and reperfusion injury via anti-inflammatory and anti-oxidative stress effects in rats. Am. J. Chin. Med..

[58] Jin X., Zhang Z., Sun E., Li S., Jia X. (2012). Statistically designed enzymatic hydrolysis of an icariin/β-cyclodextrin inclusion complex optimized for production of icaritin. Acta Pharm. Sin. B.

[59] Liu R., Li A., Sun A., Cui J., Kong L. (2005). Preparative isolation and purification of three flavonoids from the Chinese medicinal plant *Epimedium koreamum* Nakai by high-speed counter-current chromatography. J. Chromatogr. A.

[60] Xiong W., Ma X., Wu Y., Chen Y., Zeng L., Liu J., Sun W., Wang D., Hu Y. (2015). Determine the structure of phosphorylated modification of icariin and its antiviral activity against duck hepatitis virus A. BMC Vet. Res..

[61] Ito Y., Hirayama F., Suto K., Sagara K., Yoshida T. (1988). Three flavonol glycosides from *Epimedium koreanum*. Phytochemistry.

[62] Cui L., Na M., Oh H., Bae E.Y., Jeong D.G., Ryu S.E., Kim S., Kim B.Y., Oh W.K., Ahn J.S. (2006). Protein tyrosine phosphatase 1B inhibitors from Morus root bark. Bioorg. Med. Chem. Lett..

[63] Li T., Zhang X.D., Song Y.W., Liu J.W. (2005). A microplate-based screening method for alpha-glucosidase inhibitors. Chin. J. Clin. Pharmacol. Ther..

[64] Lineweaver H., Burk D. (1934). The determination of enzyme dissociation constants. J. Am. Chem. Soc..

[65] Dixon Á. (1953). The determination of enzyme inhibitor constants. Biochem. J..

[66] Cornish-Bowden A. (1974). A simple graphical method for determining the inhibition constants of mixed, uncompetitive and non-competitive inhibitors. Biochem. J..

[67] Bernstein F.C., Koetzle T.F., Williams G.J., Meyer E.F., Brice M.D., Rodgers J.R., Kennard O., Shimanouchi T., Tasumi M. (1977). The protein data bank: A computer-based archival file for macromolecular structures. Eur. J. Biochem..

[68] Berman H.M., Battistuz T., Bhat T.N., Bluhm W.F., Bourne P.E., Burkhardt K., Feng Z., Gilliland G.L., Iype L., Jain S. (2002). The protein data bank. Acta Crystallogr. D Biol. Crystallogr..

[69] Wiesmann C., Barr K.J., Kung J., Zhu J., Erlanson D.A., Shen W., Fahr B.J., Zhong M., Taylor L., Randal M. (2004). Allosteric inhibition of protein tyrosine phosphatase 1B. Nat. Struct. Mol. Biol..

[70] Pettersen E.F., Goddard T.D., Huang C.C., Couch G.S., Greenblatt D.M., Meng E.C., Ferrin T.E. (2004). UCSF Chimera—A visualization system for exploratory research and analysis. J. Comput. Chem..

